# Clinical problems due to encephalomyocarditis virus infections in two pig herds

**DOI:** 10.1186/s40813-016-0036-z

**Published:** 2016-08-07

**Authors:** Klaas Vansteenkiste, Tommy Van Limbergen, Ruben Decaluwé, Marylène Tignon, Brigitte Cay, Dominiek Maes

**Affiliations:** 1 Department of Reproduction, Obstetrics and Herd Health, Ghent University, Ghent, Belgium; 2 Veterinary and Agrochemical Research Centre, Machelen, Belgium

**Keywords:** Encephalomyocarditis virus, EMCV, Case report, Sudden death, Suckling and nursery pigs

## Abstract

**Background:**

Infections with encephalomyocarditis virus may cause myocarditis and sudden death in young pigs and reproduction disorders in sows. The presence of encephalomyocarditis virus infected rodents is considered a major risk factor for transmission of the virus to pigs. There is currently no effective treatment. Tightening up biosecurity, applying effective rodent control and reducing stress are the main control measures.

**Case presentation:**

Two farrow-to-finish herds suffering from problems with sudden death are presented. In herd A, suckling piglets from 3 to 12 days old were dying acutely whereas in herd B, piglets at the end of the nursery period (8–10 weeks) were showing identical problems. A presumptive diagnosis of encephalomyocarditis virus infection was made because typical lesions were observed in some of the affected pigs. These lesions were not always present in pigs dying acutely or in some cases the lesions were very subtle. Therefore other causes had to be ruled out based upon clinical history, clinical signs and diagnostic tests. A conclusive diagnosis was finally established by showing encephalomyocarditis virus in heart tissue using conventional gel-based polymerase chain reaction tests. The real-time PCR test that gave initially negative result was further optimized to avoid false negative results.

**Conclusions:**

Typical lesions are not always present in piglets infected with encephalomyocarditis virus, indicating the importance of examining multiple animals. Problems in suckling piglets may occur in affected herds without reproductive problems in sows. Transmission routes of EMCV in swine are not fully understood. A stand-empty period following thorough cleaning and disinfection is recommended for controlling EMC virus infections.

## Background

The encephalomyocarditis virus (EMCV) is a non-enveloped positive single-stranded RNA virus. The EMCV serotype is a member of the *Cardioviridae* genus of the *Picornaviridae* family [1]. The virus is very resistant and stable in a wide pH-spectrum. The virus may be inactivated in water with 0.5 ppm chloride, iodine and mercury(II)chloride [2]. Infections with EMCV are demonstrated in multiple species, including humans [3]. The natural reservoir consists of rodents such as rats and mice; these usually do not show clinical signs [4]. They may shed the virus via feces and urine. Pigs can be infected by contact with contaminated feces and urine, or by intake of dead rodents. The presence of EMCV infected rodents on a pig herd is the most important risk factor (OR 8.3) for developing a clinical outbreak of EMCV [5]. Whereas horizontal transmission is common in rodents [6], it is not considered to be very effective in pigs. Horizontal transmission is possible [7] but less likely [8] because of the short viremic period and the rapid progress of disease.

In pigs the main lesion is an acute myocarditis, especially younger piglets are affected [9]. The disease is often peracute in younger pigs and sudden death follows cardiac failure. Pigs dying acutely show tachycardia, fever and nausea. Circulatory collapse, hypothermia and cyanosis may precede death [10].

Sows infected during gestation usually do not show clinical signs of illness. However, EMCV in sows may cross the placenta and cause reproduction disorders such as stillbirth, prenatal birth, weak born piglets, mummies, neonatal death and abortion [11]. Abortion and mummified piglets at birth mainly occur when infection takes place during mid and late gestation [12].

The most eye-catching lesions in the acute phase are multiple circular to linear foci in the myocardium, mainly the right ventricle. Histologically these foci consist of necrosis with lysis of the sarcoplasm and mineralization. In some cases, there is lymphocytic infiltration in the myocardium [13]. Excessive pleural, peritoneal and pericardial fluid can also be found. Edema of the lungs and hyperemia of the tonsils can be present [7]. Histopathological changes may also include infiltration of inflammatory cells in the meninges, perivascular cuffing in the cerebral cortex and hippocampi and neuronal degeneration and gliosis [13].

A recent history of reproductive disorders in sows combined with the occurrence of sudden death in weaners and/or fatteners can be a first indication of EMCV infection. Clinical history, necropsy findings and histopathological examination are useful for diagnosis but they are not conclusive. To establish a conclusive diagnosis, the virus must be isolated from infected tissue (heart, spleen, blood, lung), fetuses, feces or nasal secretions. This is possible by inoculating tissue or secretions in cell cultures such as BHK-21, HeLa and Vero [2, 14, 15]. Cell cultures showing cytopathic effect must be examined by virus identification tests. Most commonly a virus neutralization test (VN) with a specific EMCV antiserum is used [11]. The identification of EMCV is also possible by using a reverse transcription polymerase chain reaction (RT-PCR) after isolation in cell cultures or directly on heart, lung, fetus or blood samples [16, 17]. Several serological tests such as ELISA, agar gel immunodiffusion and VN have been described to demonstrate antibodies against EMCV [13]. VN is most commonly used and titers of ≥16 are conclusive [18].

Unfortunately, there is currently no effective treatment against EMCV infection. When facing a clinical outbreak on a pig herd, mortality can be reduced by preventing excitation and stress. Deficiencies in vitamin E (vit E) and/or Selenium (Se) are the main cause for developing mulberry heart disease, which is clinically similar to EMCV, especially in young, fast growing pigs [19]. At necropsy, damage of the myocardium is present with foci of necrosis. For this reason vit E and Se are often supplemented when EMCV is diagnosed on a pig herd.

There are currently no vaccines available against EMCV in Europe. An effective rodent control program, cleaning and disinfection are important preventive measures. Iodine and chloride are efficacious against EMCV [2] and are commonly used in commercial disinfection products.

## Case presentation

In this case report two pig herds with problems of EMCV infection will be discussed.

### History

#### Herd A

**Fig. 1:**
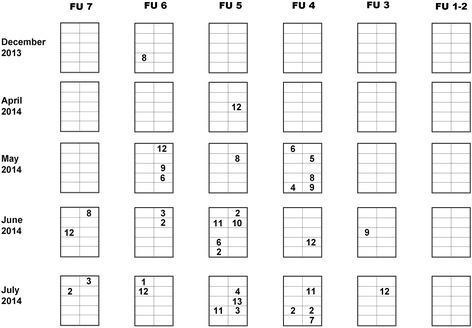
Map of the farrowing units per group of sows. Numbers represent typical piglets dying acutely per litter; each row represents one group (month); each column represents one farrowing unit (FU)

The first herd, herd A, suffered from acute and elevated mortality of suckling piglets, 3 to 12 days old. Piglets started to shiver and died within a quarter of an hour. No other clinical signs were present. In some cases, piglets became pale before dying. The mortality rose up to 22 %, normally this farm had a mortality rate of less than 12 %. Only a few extra deaths occurred in pigs older than 12 days. In Fig. 1, the dates, locations and mortality of the typical cases are displayed by the farmer. Often different piglets of a litter were affected and in many cases, these were litters with stronger and bigger piglets. Problems were not present in farrowing house 1–2. Sows were not showing any clinical signs. No abnormalities were observed for gestation length, the farrowing process and colostrum and milk production. Piglets were intensively transferred from one sow to another during the first four days after birth (up to 30 %), but piglets were never moved to an affected litter.

#### Herd B

The second herd, herd B, had problems with acute mortality in piglets from 8 to 10 weeks of age (20-25 kg). Affected pigs showed severe dyspnea prior to dying. In most cases dead animals were found without preceding clinical signs. Similar as in herd A, problems were clustered. Severe problems were present in some pens whereas no affected pigs were present in other pens. Most problems were present in pens with weaker piglets. Total mortality rate in the nursery exceeded 30 %. Sows did not show any problems. No abnormalities were observed in the farrowing period. A description of the two case herds is presented in Table 1.

**Table 1:** Overview of the characteristics of the two case herds

	Herd A	Herd B
Clinical signs	Acute mortality, shivering	Acute mortality, severe dyspnea
	preceding death	prior to death
Affected production stage	Farrowing unit	Nursery unit
Age of affected pigs	3–12 days	8–10 weeks
Mortality rate in affected production stage (%)	22	30–40
Clinical signs in sows	None	None
Duration of problems	8 months	5 weeks

### Clinical history

#### General herd characteristics

An overview of general characteristics of herd A and B is summarized in Table 2.

**Table 2:** General information of case herds A and B

	Herd A	Herd B
Herd size (number of sows)	300 sows	300 sows
% of piglets raised as fattening pigs on the same site	90	30
Type of batch farrowing system for sows	4-week	3-week
Age of weaning (weeks)	3	4
Breed of sows	Topigs	Danbred
Breed of boar for artificial insemination	Belgian Piétrain	Belgian Piétrain
Purchase of breeding gilts	No	No
Floor structure	Fully slatted	Fully slatted

*BMS* Batch Management Production System

#### Routinely performed interventions and treatment of piglets

Routinely performed interventions and treatment of piglets are summarized in Table 3.

**Table 3:** Routinely performed interventions and treatment of piglets on herd A and B

Age (in days)	Intervention	Age (in days)	Intervention
Herd A		Herd B	
1	Teeth clipping	1	Teeth clipping
2–3	Iron (IM)	3–4	Iron (IM)
	Tulathromycin (IM)		Tulathromycin (IM)
	Tail docking		Toltrazuril (PO)
5–7	Surgical castration	8	Surgical castration
	*M. hyponeumoniae* vaccination		Amoxicilin (IM)
	Toltrazuril (PO)		*M. hyopneumoniae* vaccination
		18	Porcine circovirus type 2 vaccination

*IM* intramuscularly*, PO* per os

#### Drinking water

##### Herd A

All animals in the herd were supplied with the same drinking water. It was a combination of ground water and rainwater. Water was guided through a sand filter and was disinfected before problems appeared. Since problems appeared, acidification took place by using a blend of organic acids (Acilux®, Jodoco, Belgium). In early July 2014 water was analyzed. Most of the parameters were within normal values, except for total plate count at 22 °C (125000 colony forming units (CFU)/ml), intestinal enterococci (3300 CFU/100 ml) and bicarbonates (346 mg/l).

##### Herd B

All compartments of the herd were supplied with public water. Neighboring pens shared drinking nipples. No analysis of the drinking water was performed.

#### Feed

##### Herd A

Sows were fed with a commercial feed. From insemination until farrowing, a gestation feed was used (100 international units (IU)/kg vit E). From farrowing until weaning, lactation feed was used (150 IU/kg vit E). Because of the problems, the sows were supplemented (2 ml/sow) from 3 days before farrowing until 3 days after farrowing a mix containing vit E (150000 mg/l) and Se (50 mg/l).

##### Herd B

Similar as in herd A, commercial feed was used on herd B. In the farrowing house, piglets were already fed a solid pre starter feed (150 mg/kg vit E; 0,32 mg/kg Se) a few days before moving to the nursery. In the nursery, starter feed was given until 12 weeks of age (80 mg/kg vit E; 0.4 mg/kg Se). Because of the sudden death problems in the nursery, Liquid E+ SE® (Panagro, Belgium)(200 g/l vit E;0.05 g/l Se) was supplemented, without success. Similar as with the drinking nipples, the feeding troughs were shared between adjacent pens. Although pigs of adjacent pens could have nose-nose contact, it was possible that only one pen was affected.

#### Cleaning and disinfection

##### Herd A

Cold water was used for cleaning the pens. Pens were first soaked with a commercial product (Logic Ultra Cleaner®, Agro Logic, Belgium). Dirt was removed by using water under high pressure (220 bar). The applied disinfection product (Hi logic®, Agro Logic, Belgium) contained chloride, which is active against EMCV. Farrowing houses were not rinsed afterwards. Ventilation was set at 100 % for 24 h to improve drying. Farrowing house 1–2 (one compartment) was left empty for 5–6 days because it was used for pigs that were weaned earlier than the entire group. The other compartments were left empty for only one day which is common in a 4-week batch system. The drinking troughs were made of porous material and hence, they were difficult to clean.

##### Herd B

The compartments were soaked with a commercial product (Topfoam®, Schippers, Netherlands). Cold water under high pressure (200 bar) was used for cleaning. Disinfection was done with Virocid® (CID Lines, Belgium). Nothing was utilized to speed up the drying process. The stand-empty period for the farrowing, nursery and fattening units was 1, 3 and 1 week(s) respectively. Pens with bigger and stronger pigs were not routinely cleaned and disinfected, except once when the problems started.

#### Rodent control

In both herds, a professional company was responsible for the rodent control. In herd A no excessive trails such as feces, damage to insulation and corpses of rat and mice were observed. In herd B, trails, such as damage to insulation and presence of rat feces were visible. In addition, feeding troughs were accessible for rodents from the top, windows of the stable were not covered with nettings and cats could enter the building.

### Analyses and results

#### Herd A

**Fig. 2:**
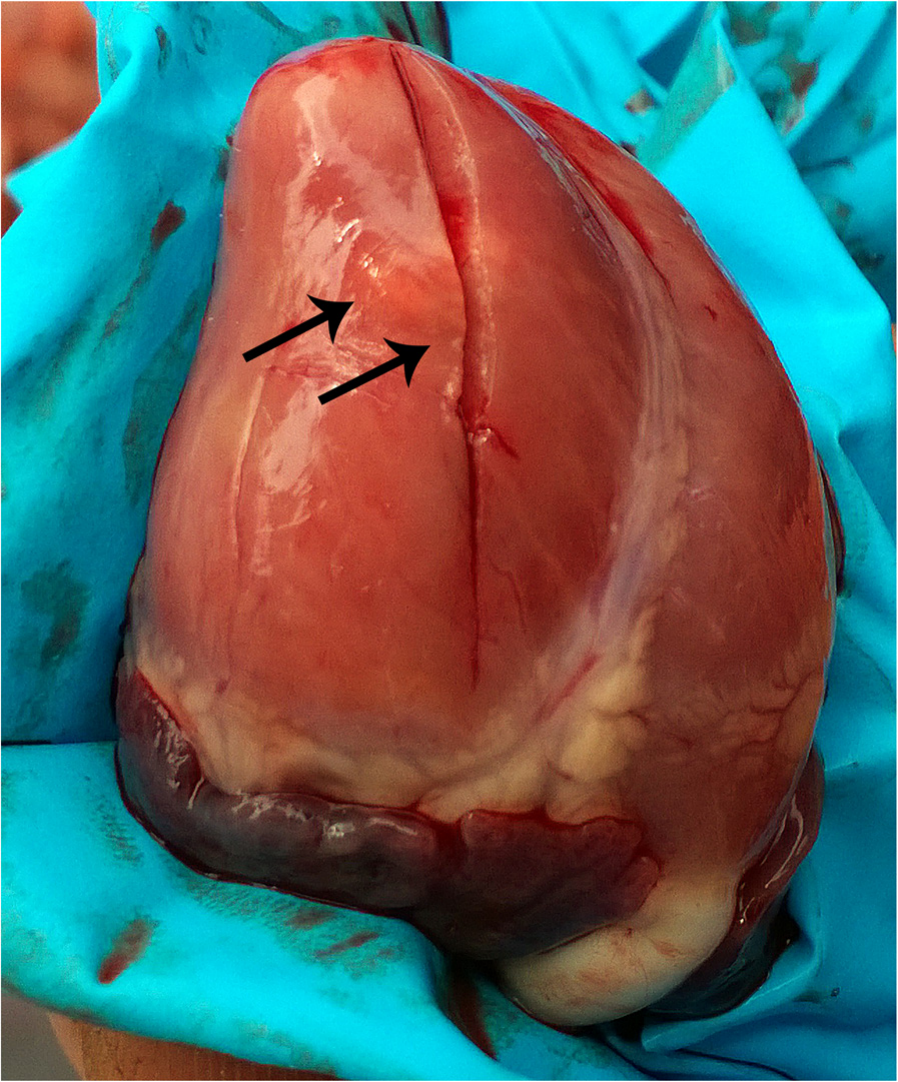
Heart of a piglet showing a subtle EMCV lesion. Arrows indicating subtle necrosis of the myocard

The examinations performed in herd A are summarized in Table 4. The first two episodes of sudden death problems in 3 day old piglets (December 2013 and January 2014) were not thought to be related with EMCV because no signs indicative for EMCV were observed. Instead *E. coli* was suspected as the causative pathogen. However, in January 2014 no virulence factors for *E. coli* (F4, F5, F6 or F41 adhesion factors; LT, STa and STb toxins) were found. An intoxication appeared to be unlikely due to the clustering of problems. Dead piglets did not appear to have been crushed by the sow and they were often heavier than the remaining pigs. The microclimate appeared to be good for the piglets. The first examination suggesting EMCV took place in June 2014 and was performed by the herd veterinarian. Upon necropsy of a 5 day old piglet, a very subtle lesion on the heart compatible with EMCV was observed (Fig. 2).

**Table 4:** Overview of the different examinations performed on herd A

Date	Examination (number of piglets)	Main finding
December 2013	Necropsy^b^ (2)	Indicative for *Escherichia coli* septicemia
	Histology heart^b^	Degeneration of myocardium fibres
	Anaerobic culture duodenum^b^	Negative
	Aerobic culture jejunum^b^	Positive (*E. coli*)
January 2014	Necropsy^b^ (5)	No abnormalities
	Histology heart^b^	Moderate degeneration myocardium fibres
	Anaerobic culture duodenum^b^	Negative
	Aerobic culture jejunum^b^	*Staphylococcus* spp. en *Streptococcus* spp
	Mesenterial lymph nodes^b^	*Escherichia coli*
	Serotyping *Escherichia coli* ^c^	No virulence factors found
June 4 2014	Necropsy^a^ (2)	Subtle EMCV-lesion on heart muscle
	Necropsy^b^ (14)	None
	Histology^b^	Myocarditis and myocardnecrosis suggesting EMCV
June 6 2014	Necropsy^b^ (17)	White foci in myocardium suggesting EMCV
	Histology^b^	Necrosis in myocardium suggesting EMCV
August 12 2014	Necropsy^b^ (^d^)(2)	None
August 13 2014	PCR EMCV heart samples June 2014^c^	Negative

^a^ Herd veterinarian; ^b^ Animal Health Care Flanders; ^c^ Veterinary and Agrochemical Research Centre, Brussels; ^d^ no typical piglets available

Necropsies that followed were also suggestive to an EMCV infection except the one on August 12 probably because no typical piglets were available at that time. The hearts of two necropsied piglets were submitted to a real-time TaqMan PCR assay targeting a 80 bp segment in the 3D polymerase region. No conclusive diagnosis could be made as PCR results turned out negative. Pathognomonic EMCV lesions were not present or appeared to be very subtle.

In addition to these examinations, all individual sow reproduction data were analyzed in August 2014 whether parity, number of live born piglets, number of stillborn piglets, and number of mummies were associated with the problems. None of these factors turned out as a potential risk factor.

#### Herd B

In herd B two main examinations took place, summarized in Table 5. Immediately EMCV was suspected in this case although other diseases such as edema and Glässers disease and streptococcal meningitis could cause sudden death in the nursery. However clinical nervous signs and slower progression of disease would be expected. A real time PCR was performed to demonstrate EMCV but similar as in herd A the result was negative. Because all PCR results were negative, the hypothesis rose that these farms were facing an uncommon variant of EMCV. The samples from farm A and B were submitted to two individual gel-based real time PCR assays targeting either a 903 bp region in the capsid coding region using primer CCR-L and CCR-R [20] or a 268 bp region in the 3D polymerase gene using primers P1 and P2 [21]. PCR results turned out to be positive with the gel-based real time PCR. Sequencing of the 3D pol segment did not indicate the presence of mutation in the genetic sequence susceptible to alter the hybridization of the primers or the probe in the real-time PCR assay. It was further demonstrated that amplification with the real-time PCR primers occurred but detection with the Taqman probe failed. As a consequence, the real-time PCR test was adapted with replacement of the Taqman probe by SYBR® Green dye (Roche diagnostics, Belgium) in order to improve pathogen detection.

**Table 5:** Overview of the different examinations performed on herd B

Date	Examination (number of piglets)	Main findings
August 2014	Necropsy^a^ (7)	Multiple small white foci myocardium suggesting EMCV
	Histology^a^	Multifocal, expanded zones with neutrophils often with necrosis of myocardium fibres
	PCR EMCV^b^	Negative
September 2014	Necropsy^c^ (2)	Macroscopic view compatible with EMCV
	Histology^c^	No indicative signs for EMCV

^a^ Animal Health Care Flanders; ^b^ Veterinary Agrochemical Research Centre, Brussels; ^c^ Ghent University, Department of Pathology, Bacteriology and Poultry Diseases

### Recommendations

Control of EMCV infections is mainly focused on preventing EMCV to enter the pig herd and to prevent transmission within the herd and hence internal and external biosecurity play a major role in controlling this disease.

#### Herd A

Herd A was advised to pay more attention to cleaning and disinfection, especially the feeding troughs of the sows and the drinking troughs of the piglets. A longer stand-empty period of the compartments had to be practiced. As mentioned before, herd A produced according to a 4-week batch system and farrowing houses were continuously in use, making it rather impossible to implement this measure. Furthermore, the farmer was advised to reduce stress and excitation as much as possible e.g. limiting moving piglets from one sow to another, not performing teeth clipping and washing sows for the time being. The farmer refused to drop all preventive treatments of the piglets because he expected a lot of problems afterwards. Piglets were still being treated against coccidiosis, vaccinated against *Mycoplasma hyopneumoniae,* tails were docked and ear tags were placed. Feeding practices, light scheme and climate conditions were not altered. Pets such as cats and dogs were not allowed to enter the stables anymore. Because of insufficient quality of the water, water from the public supply was provided to the next group.

#### Herd B

The farmer of this herd was advised to increase the overall biosecurity level of his herd. Examples of this advice were: providing boots and clothes for visitors, installing disinfection baths, following logical walking lines, color codes for material used per age group, removing cadavers as soon as possible, etc… Interventions inducing stress or excitation were reduced as much as possible. A longer stand-empty period after disinfection and cleaning of the rooms was practiced. Furthermore, a more stringent rodent control was implemented by a professional company. Possible access routes (windows, ventilation gaps in doors) for rodents were closed including the top of feeding troughs in the nursery. Similar as in herd A, pets were denied access to buildings.

### Final outcome

#### Herd A

After implementing all recommendations no EMCV outbreak has occurred until the time of submitting this paper. Only one piglet was suspected of EMCV by the farmer, but no diagnostics were performed. To avoid any risk, the herd was advised to utilize water from the public supply for next groups as well. After 3 months water was switched back to rainwater and ground water without negative consequences.

#### Herd B

After the two suspicious cases, no more pigs with typical EMCV signs had died. It seemed the situation was normalized on this herd.

### Discussion

In these cases, two different age categories, suckling piglets and nursery pigs, were affected by EMCV. The mortality rates of 22 % and 30–40 % for herd A and B respectively caused serious economic damage to both herds which imposed immediate action. Treatment of piglets in farrowing units may cause stress and may play a role in developing any kind of disease. Indeed, in herd A, the affected piglets were 3–12 days old. Stress, caused by the multiple interventions mentioned in Table 3, may have played a role in developing EMCV problems. As observed on these farms sudden death related to EMCV may occur in the absence of reproductive disorder.

Affected piglets in herd A and B were clustered i.e. some litters or pens were affected whereas others were not, although pigs of adjacent pens could have direct nose-nose contact. This supports earlier findings that horizontal transmission in swine is not very effective [8]. This could also be an indication that transmission of EMCV between pigs is more effective via feces than via nasal secretions. The infection pattern on these herds (i.e. clustering) however, could be explained by multiple introductions because of the many infected pens and the low basic reproduction ratio (R_0_) between pigs within pens [22] described by Kluivers et al. (2006). The most convenient cause of multiple introductions in this case would be rodents. The low R_0_ could also explain the die out of EMCV problems in herd A and B after implementing a more stringent rodent control. Since infected pigs are able to excrete the virus, feces may play a role in transmission. Contact with feces on herd A and B is minimal due to fully slatted floors. Manure coming up through the slatted floor would consequently be a potential risk for infection but Maurice et al. (2007) found this event to be protective (OR = 0.11) against EMCV infection [5]. The authors suggested this protective effect was observed because pigs acquired immunity after they had contact with a low dose of EMCV in manure. This seems indeed controversial as feces and consequently manure could also be considered as sources of infection. Alternatively, the flow of manure up through the slatted floor could have resulted in a decrease in rodents (unable to walk on crusts in the manure pit, drowning) and thereby protected against EMCV. On herd A, piglets of 3 days old were already affected which supports the hypothesis of horizontal transmission because they didn’t use drinking nipples or feeding troughs yet. Horizontal transmission between pigs remains a concern, therefore it is recommended to separate different age groups, limit movements of animals, apply a good quarantine management and general biosecurity principles. Further research on transmission patterns is warranted.

Typical EMCV lesions were not found during the initial stages of the problems and/or were very subtle on herd A. This is uncommon in an EMCV outbreak and results in a more difficult diagnosis. In other cases where lesions are commonly present, diagnosis is rather straightforward. Possibly the disease progressed that quickly so that no macroscopically visible lesions developed. It was thereby important not to exclude EMCV from the differential diagnosis. As a consequence, practitioners should clinically examine as many pigs as possible to avoid missing out lesions on necropsy. White foci of necrosis on the myocardium are pathognomonic. If no typical lesions are observed or in order to establish a conclusive diagnosis, the practitioner should submit piglets or hearts to a diagnostic laboratory in order to demonstrate the virus via identification tests mentioned in the background section. These tests may differ among laboratories. In this case real time PCR was performed. After several real time PCR tests turned out negative due to quality of the test, the technique used by the veterinary and agrochemical research centre, was optimized with success.

Supplementation with vit E and Se failed to reduce EMCV problems probably because the vit E and Se levels in standard feed were sufficient. After the change to drinking water from the public supply in herd A no problems occurred in the following batches. It is not known whether both were related, as switching back to ground and rain water three months later, no clinical EMCV problems were observed. Practicing a stand-empty period for a few days in the compartments following cleaning and disinfection could reduce the presence of EMCV in the environment because farrowing unit 1–2 in herd A, which was consistently left depopulated for 5-6 days, did not show problems.

## Conclusions

EMCV infection caused sudden death problems in the suckling and nursery pigs in these two herds, without causing reproductive disorders in sows. Eventually, EMCV was confirmed by real time PCR on herd B. Pathognomonic lesions were not always present in pigs suffering EMCV which was uncommon. Clustering of affected pigs within the same litter (farrowing unit) or pen (nursery unit) was observed.

The exact transmission routes are not fully understood but this case report suggests horizontal transmission is possible, consequently implementation or tightening of general biosecurity measures are recommended. Effective rodent control is considered the most important measure in controlling EMCV. Leaving compartments depopulated for a few days following cleaning and disinfection could also be helpful. Supplementing vit E and Se was not effective in reducing clinical problems.

## Abbreviations

CFU, colony forming units; EMCV, encephalomyocarditis virus; IM, intramuscularly; IU, international units; PO, per os; RT-PCR, reverse transcription polymerase chain reaction; VN, virus neutralization
